# Cholera in Pregnancy: A Systematic Review and Meta-Analysis of Fetal, Neonatal, and Maternal Mortality

**DOI:** 10.1371/journal.pone.0132920

**Published:** 2015-07-15

**Authors:** Nguyen-Toan Tran, Richard Taylor, Annick Antierens, Nelly Staderini

**Affiliations:** 1 Médecins Sans Frontières, Geneva, Switzerland; 2 School of Public Health and Community Medicine, University of New South Wales, Sydney, New South Wales, Australia; UCL Institute of Child Health, University College London, UNITED KINGDOM

## Abstract

**Background:**

Maternal infection with cholera may negatively affect pregnancy outcomes. The objective of this research is to systematically review the literature and determine the risk of fetal, neonatal and maternal death associated with cholera during pregnancy.

**Materials and Methods:**

Medline, Global Health Library, and Cochrane Library databases were searched using the key terms *cholera* and *pregnancy* for articles published in any language and at any time before August 2013 to quantitatively summarize estimates of fetal, maternal, and neonatal mortality. 95% confidence intervals (CIs) were calculated for each selected study. Random-effect non-linear logistic regression was used to calculate pooled rates and 95% CIs by time period. Studies from the recent period (1991-2013) were compared with studies from 1969-1990. Relative risk (RR) estimates and 95% CIs were obtained by comparing mortality of selected recent studies with published national normative data from the closest year.

**Results:**

The meta-analysis included seven studies that together involved 737 pregnant women with cholera from six countries. The pooled fetal death rate for 4 studies during 1991-2013 was 7.9% (95% CIs 5.3-10.4), significantly lower than that of 3 studies from 1969-1990 (31.0%, 95% CIs 25.2-36.8). There was no difference in fetal death rate by trimester. The pooled neonatal death rate for 1991-2013 studies was 0.8% (95% CIs 0.0-1.6), and 6.4% (95% CIs 0.0-20.8) for 1969-1990. The pooled maternal death rate for 1991-2013 studies was 0.2% (95% CIs 0.0-0.7), and 5.0% (95% CIs 0.0-16.0) for 1969-1990. Compared with published national mortality estimates, the RR for fetal death of 5.8 (95% CIs 2.9-11.3) was calculated for Haiti (2013), 1.8 (95% CIs 0.3-10.4) for Senegal (2007), and 2.6 (95% CIs 0.5-14.9) for Peru (1991); there were no significant differences in the RR for neonatal or maternal death.

**Conclusion:**

Results are limited by the inconsistencies found across included studies but suggest that maternal cholera is associated with adverse pregnancy outcomes, particularly fetal death. These findings can inform a research agenda on cholera in pregnancy and guidance for the timely management of pregnant women with cholera.

## Introduction

Cholera is an acute diarrheal disease caused by the bacterium *Vibrio cholerae* and transmitted through fecally-contaminated water or food, which affects children and adults [1]. Around 20% of the infected individuals develop acute, watery diarrhea, and 10% to 20% of them develop severe watery diarrhea with vomiting [2]. If these patients do not receive prompt and adequate treatment, the massive loss of fluid and electrolytes can lead to severe dehydration and death within hours. The case-fatality rate in untreated cases with severe cholera may reach 70% [3]. Rapid and intensive fluid therapy and supportive care should keep the case-fatality rate below the internationally-accepted threshold of 1% [4], and therefore avert the high mortality that cholera historically caused. WHO estimates the global disease burden to be 3 to 5 million cases and 100,000 to 100,300 deaths each year, occurring mostly in Asia and Africa, with periodic epidemics including the recent Haiti epidemic [4]. The link between cholera in pregnancy and negative childbirth outcomes has been observed since the 19^th^ century [5]. The 2010–2011 cholera outbreak in Haiti suggested again that cholera negatively impacts on pregnancy outcomes [6]. The experience of Médecins sans Frontières (MSF) treating pregnant women with cholera in Haiti underscored the need to better understand the extent that cholera affects pregnancy and pregnancy outcomes, as to inform treatment guidelines and set benchmarks for cholera-related pregnancy outcomes. However, the World Health Organization (WHO), which hosts the Global Task Force of Cholera Control, and the Center for Disease Control do not address cholera in pregnancy in their guidelines [7–11]. In the studies reviewed, evidence documenting the severity of cholera during pregnancy could not be determined, and no previous meta-analysis that estimates the extent to which cholera affects pregnancy outcomes, such as maternal, fetal, and neonatal deaths, was identified. In light of the scarcity of published evidence on the subject and the public health importance of addressing the needs of pregnant women with cholera, MSF launched this systematic research and quantitative meta-analysis with the objective to determine the risks of fetal demise and neonatal and maternal mortality in pregnant women with cholera.

## Materials and Methods

### Search strategy and selection criteria

Based on the PRISMA guidelines, a systematic review of the literature was undertaken with a search of Medline, Global Health Library, and Cochrane Library databases for articles published in any language and at any time before August 2013 with the key terms *cholera* and *pregnancy*. No other search terms were used. Bibliographies of relevant published articles were also searched. For papers not excluded based on title and abstract, studies of any design were included if they satisfied the following inclusion criteria: (i) the study involved pregnant women with cholera, (ii) the number of fetal, neonatal, and maternal deaths linked to cholera was assessed. Full text analysis was limited to studies that met the inclusion criteria.

### Data extraction

Eligible studies were selected and data abstracted by one of the authors (NTT) and reviewed by another (NS), using a pre-agreed table. Discrepancies were examined together by both authors before reaching consensus. For each study that met inclusion criteria, data were extracted on: the first author’s last name; the year of publication; the year(s) of the cholera outbreak; the country where the study was performed; the number of pregnant women with cholera; fetal deaths (defined as stillbirth or fetal loss irrespective of the pregnancy duration); neonatal deaths (defined as death of liveborn infant within the first 28 days of life); maternal deaths (defined in this instance as death of a woman while pregnant or within 42 days of termination of pregnancy); and key study features, study quality and risks of bias. All data used in this article are derived from published studies and reports. No unit record or individual data were obtained or accessed; only publicly available count data were used in calculations.

### Statistical analysis

Rates for fetal, neonatal and maternal mortality were obtained by dividing the deaths by the appropriate number of pregnant women with cholera as the denominator. The denominator for neonatal mortality was the number of women in the third trimester reduced by subtracting fetal deaths (assuming singleton pregnancies). Missing data were excluded from relevant sub-group analyses, such as pregnancies of unknown trimester.

For each study, 95% confidence intervals (CIs) for rates (or ratios) were calculated using the Poisson distribution because of small numbers and instances of zero deaths. Statistical heterogeneity was assessed using the *χ* ^*2*^ test and non-statistical heterogeneity assessed by the Higgins *I* ^*2*^ statistic. Due to statistical heterogeneity, meta-analysed pooled estimates were obtained using random-effect models and because of small number, non-linear logistic regression analysis was employed following expansion of studies into unit record files. Negative CIs were approximated to zero. The meta-analysis was performed by time period (1991–2013 and 1969–1990) based on the reported use of oral rehydration therapy (ORS) in the included studies from 1991 onward. Statistical calculations and analyses were performed in Excel spreadsheets and STATA 11 (Statacorp, College Station, Texas, USA). Outputs in meta-analyses were double checked by the same person (RT) for internal consistency. There were insufficient studies to plot a frequency distribution to investigate possible publication bias.

Risk ratio (RR) estimates were obtained by comparing fetal, neonatal and maternal mortality rates of selected studies (with more than zero deaths), with published national estimates of rates of the closest year where available (Haiti, Senegal, Peru). UN sources were used for national neonatal and maternal mortality [12,13], but no national estimates were located for periods before 1990. As there are no national estimates for first and second trimester fetal mortality, only risk ratios for third trimester fetal mortality were calculated using national estimates that defined stillbirth as third trimester fetal death [14]. RRs were also calculated from the summation of data from the four studies of 1991–2013 and corresponding national estimates. Confidence intervals (95%) for RRs were calculated from study and and national counts of numerators and denominators.

## Results

**Fig 1:**
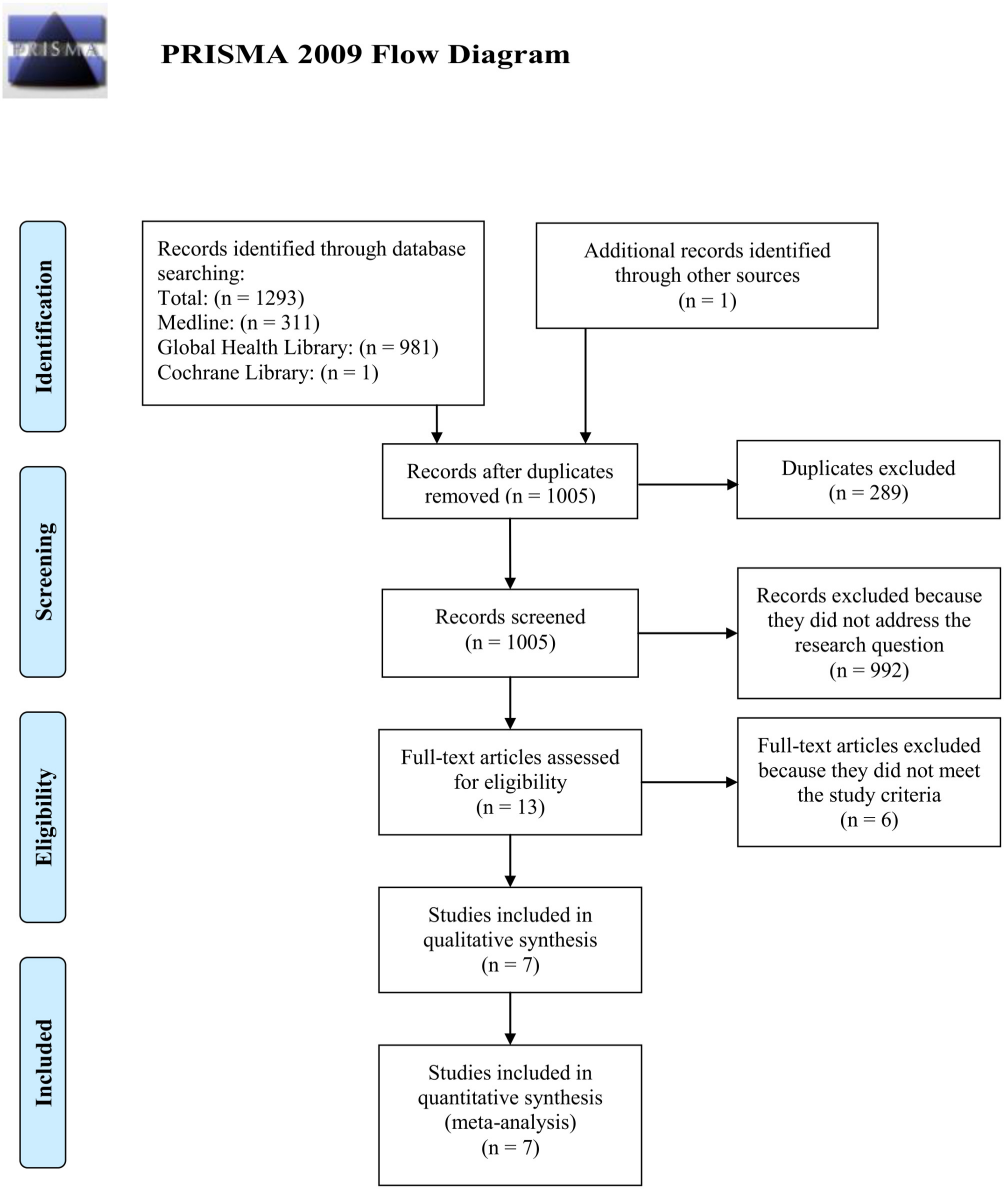
Search strategy for a systematic review and meta-analysis to determine fetal, neonatal, and maternal mortality among pregnant women with cholera.

**Fig 2:**
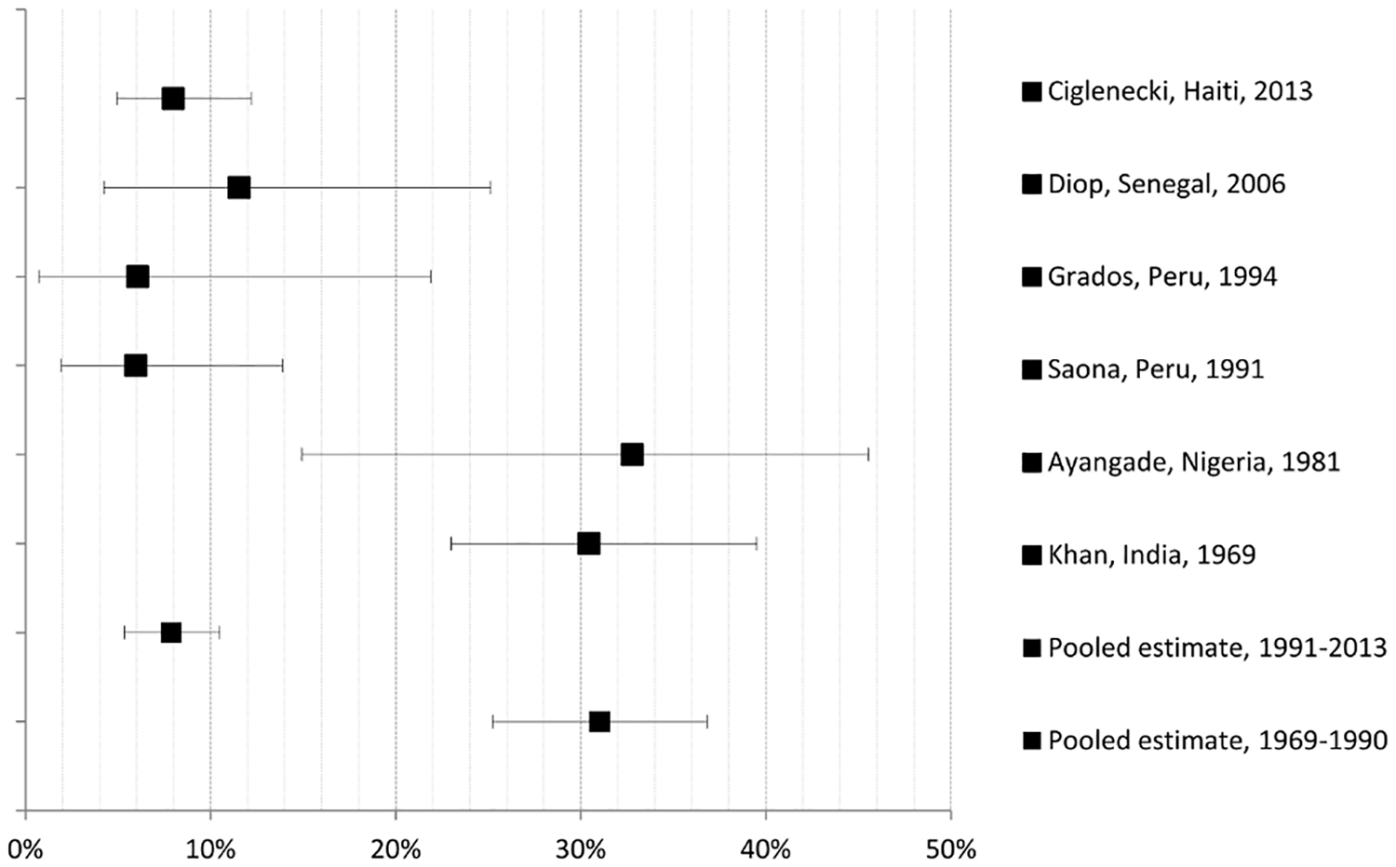
Fetal death rate (all trimesters) with maternal cholera: study and pooled estimates per 100 pregnancies with 95% confidence intervals.

**Fig 3:**
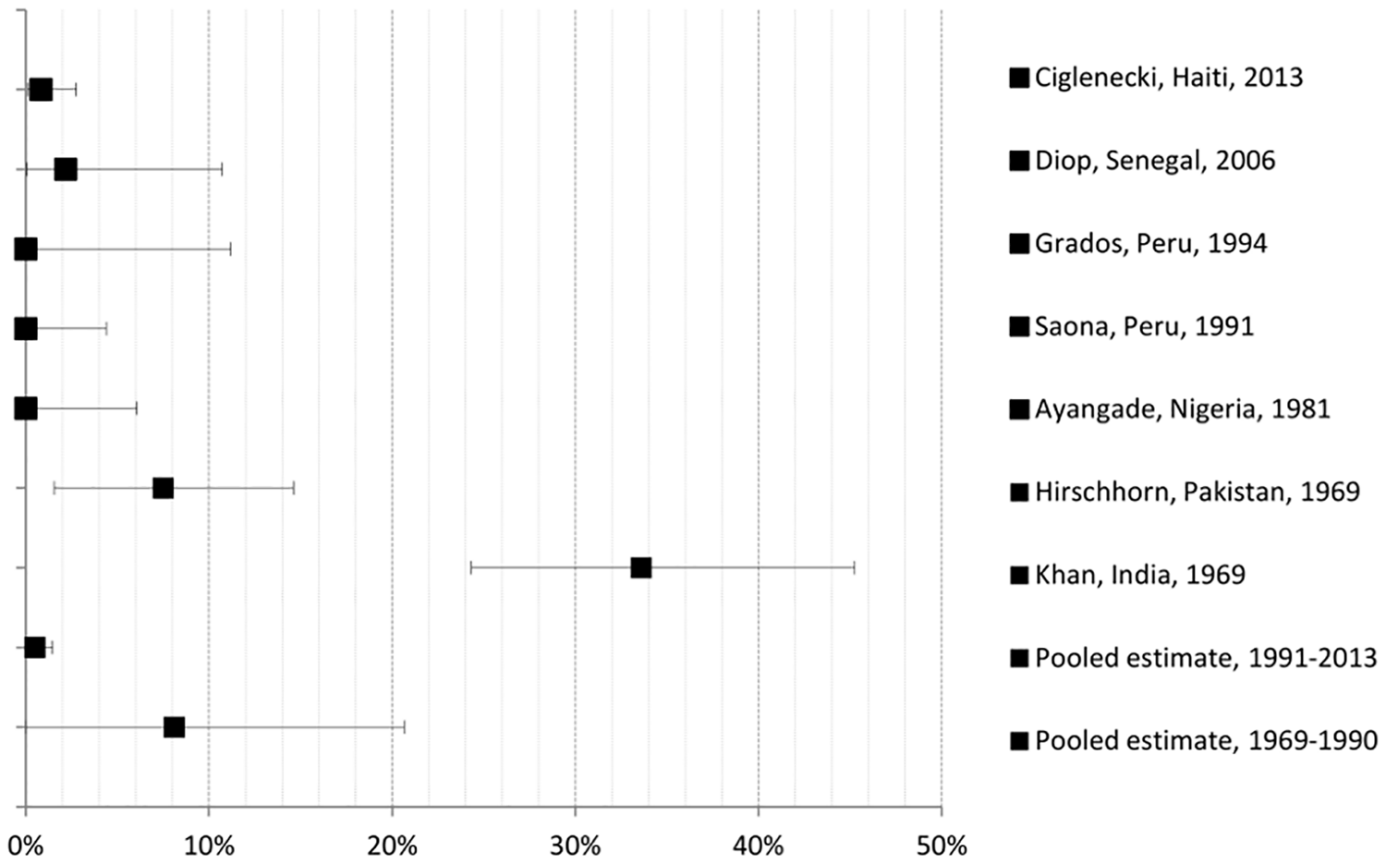
Neonatal death rate with maternal cholera: study and pooled estimates per 100 pregnancies with 95% confidence intervals.

**Fig 4:**
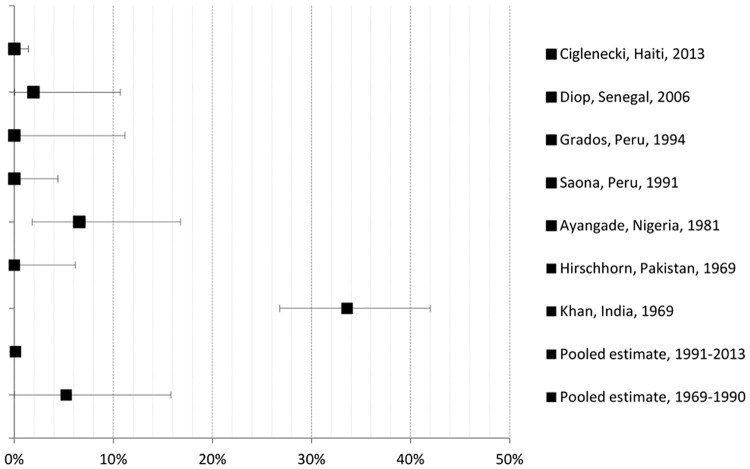
Maternal death rate with cholera: study and pooled estimates per 100 pregnancies with 95% confidence intervals.

From 1,005 potentially relevant publications, seven of these met inclusion criteria. Papers were excluded because they were duplicates, did not address the research question, or did not meet the study inclusion criteria. A flow diagram of study selection is shown in Fig 1. Seven studies were included in the meta-analysis (six cohort and one case-control study), involving together a total of 737 women (see Table 1 for general study characteristics). Studies were published between 1969 and 2013 and describe fatal pregnancy outcomes that occurred in women with cholera in health facilities and that were related to cholera outbreaks in India (1958–1963) [15], Pakistan (1962–1967) [16], Nigeria (1979–1980) [17], Peru (1991, 1992) [18,19], Senegal (2004–2005) [20], and Haiti (2010–2011) [6]. Figs 2–4 present in forest plots the fetal, neonatal and maternal death rates of each study, with pooled meta-analysed estimates, by time period.

**Table 1:** Characteristics of studies included in a systematic review and meta-analysis to determine fetal, neonatal, and maternal mortality among pregnant women with cholera.

Study, year, country	Outbreak years	No. of pregnant women admitted for cholera	Case ascertainment	Treatment received	Pregnancy outcomes	Other study characteristics
Ciglenecki [6], 2013, Haiti	2010–2011	263	Cholera suspected based on clinical presentation.	ORS, IV fluid, erythromycin. Others: patients admitted to a specialized cholera treatment unit for pregnant women; followed the dehydration classification of WHO; systematic IV glucose.	No. of fetal death: 21, No. of neonatal death: 2, No. of maternal death: 0	Used WHO dehydration assessment; cholera suspected in any patient presenting with three or more liquid stools and/or vomiting episodes in the previous 24 hours; in-depth description of interventions and outcomes; suggests that specialized multidisciplinary units could be useful in large epidemics in managing cholera in pregnancy; retrospective descriptive study; hospital-based; largest cohort studied to date; statistical analysis of risk ratios for fetal death with control for confounding factors.
Diop [20], 2007,Senegal	2004–2005	52	First cholera case confirmed by laboratory test; other cholera cases suspected based on WHO case definition.	ORS, IV fluid, various regimens of antibiotics but not systematically for all patients. Others: followed the dehydration classification of WHO.	No. of fetal death: 6, No. of neonatal death: 1, No. of maternal death: 1	Used WHO case definition and dehydration assessment; no in-depth description of maternal death; limited description of client profiles, treatment and outcomes; no mention of indication for antibiotherapy, why different regimens of antibiotics, indication for IV glucose; retrospective descriptive study; hospital-based; small sample; limited statistical analysis.
Grados [19], 1994, Peru	1992	33	A few cases confirmed by laboratory test; the others were suspected based on clinical presentation.	ORS, IV fluid. Antibiotics: not mentioned.	No. of fetal death: 2, No. of neonatal death: 0, No. of maternal death: 0	Idiosyncratic definition of tachycardia (< 90 mmHg) and mean arterial pressure; retrospective descriptive study; hospital-based; very small sample; statistical analysis with multiple regression to establish association between different variables, such as association between degree of dehydration and patient’s signs and symptoms.
Saona [18], 1991, Peru	1991	84	Cholera suspected based on clinical presentation.	ORS, IV fluid. Various regimens of antibiotics but not systematically for all patients.	No. of fetal death: 5, No. of neonatal death: 0, No. of maternal death: 0	No dehydration categorization; group disaggregation for comparison purpose; retrospective descriptive study; hospital-based; small sample; very limited statistical analysis.
Ayangade [17], 1981, Nigeria	1979–1980	61	Cholera suspected based on clinical presentation.	ORS: not mentioned. All patients received IV fluid and tetracycline.	No. of fetal death: 20, No. of neonatal death: 0, No. of maternal death: 4	Used a system of “comprehensive case finding”, allowing to determine total number of cases beyond the hospital admission cases; included case follow up; limited description of cholera treatment; unclear how pregnancy was diagnosed in month 1 and 2; questionable comparison group for cholera survival: pregnant women vs. < 15-year old or non-pregnant women, without adjusting for confounding factors; prospective descriptive study; small sample; limited statistical analysis.
Hirschhorn [16], 1969, Pakistan	1962–1967	60 (first-trimester pregnancies excluded)	All cases confirmed by laboratory test.	ORS: not mentioned, IVF. Tetracycline or chloramphenicol but not systematically.	No. of fetal death: 20, No. of neonatal death: 3, No. of maternal death: 0	Disaggregated cases into four comparison groups, including a control group; measurement of plasma protein concentration on admission and exit allowed quantitative assessment of dehydration; additional investigation: negative culture of placenta, fetal blood and amniotic fluid on 4 autopsies; retrospective case-control study; hospital-based; small sample; simple statistical analysis.
Khan [15], 1968, India	1958–1963	184	Not mentioned.	Not mentioned.	No. of fetal death: 56, No. of neonatal death:43, No. of maternal death:47	Non-interventional; description of evolution of disease without treatment and report of causes of maternal death; assessed abdominal girth and diarrhea severity; prospective descriptive study; hospital-based; larger sample; limited statistical analysis.

### Fetal mortality (Fig 2)

In 1991–2013, fetal death rates associated with symptomatic maternal cholera ranged from 6.0% (95% CIs 1.9–13.9) in Peru/Saona (1991) to 11.5% (95% CIs 4.2–25.1) in Senegal (2006). In 1969–1990, fetal death rates were around 30%, with 32.8% in Nigeria (95% CIs 20.0–50.6), 33.3% in Pakistan (95% CIs 20.4–51.5), and 30.4% in India (95% CIs 23.0–39.5). Pooled meta-analysed fetal death rates by period displayed a statistically significant difference (p <0.0001) between 1991–2013 (7.9%, 95% CIs 5.3–10.4) and 1969–1990 (31.0%, CIs 25.2–36.6). Within both the 1991–2013 and 1969–1990 groups of studies, there was no statistical heterogeneity based on *χ* ^*2*^ (p<0.05), and there was minimal non-statistical heterogeneity according to Higgins *I* ^*2*^. Further pooled analysis of the 1991–2013 studies did not suggest a difference in risk of fetal death by trimester: first trimester: 6.2%, 95% CIs 0.9–11.4; second trimester: 9.1%, 95% CIs 3.5–14.6; third trimester: 6.7%, 95% CIs 0.7–12.8. When compared with national stillbirth estimates, results indicate an elevated relative risk (RR) of third-trimester fetal death for the cohorts in Haiti (2013) of 5.8 (95% CIs 2.9–11.4 and p<0.0001). Although the RR was elevated in Senegal (2006) at 1.8 (95% CIs 0.3–10.4), the p-value was not significant (p = 0.5295), which was also the case in Peru (1991) with a RR of 2.6 (95% CIs 0.5–14.9 and p = 0.3227). For all four studies of 1991–2013, the RR is 3.83 (95% CIs 2.08–7.05) which is significant at p<0.0001.

### Neonatal mortality (Fig 3)

In 1991–2013, the neonatal death rates associated with symptomatic maternal cholera ranged from 0.0% (95% CIs 0.0–4.4) in Peru/Saona (1991) to 2.2% (95% CIs 0.1–10.7) in Senegal (2006). In 1969–1990, it ranged from 0.0% (95% CIs 0.0–6.1) in Nigeria (1990) to 33.6% (95% CIs 24.3–45.3) in India (1969). The pooled neonatal death rate for 1991–2013 was 0.8% (95% CIs 0.0–1.6), and 6.4% (95% CIs 0.0–20.8) for 1969–1990, which is significantly different at p = 0.021. Studies in the 1991–2013 period showed no statistical heterogeneity according to *χ* ^*2*^ (p<0.05), and the Higgins *I* ^*2*^ for non-statistical heterogeneity was correspondingly minimal; whereas for the 1969–1990 studies, there was statistical heterogeneity (*χ* ^*2*^ (2df) = 40.3, p<0.001) and considerable non-statistical heterogeneity (Higgins *I* ^*2*^ = 95%). When compared with national estimates, there was no increased RR of neonatal death from maternal cholera for the cohorts in Haiti (RR 0.3, 95% CIs 0.1–1.2 and p = 0.1160) or Senegal (RR 0.6, 95% CIs 0.1–3.6 and p = 0.6512). For all four studies of 1991–2013, the RR is 0.28 (95% CIs 0.10–0.84), which is significant at p = 0.0290.

### Maternal mortality (Fig 4)

In 1991–2013, the maternal death rates associated with symptomatic cholera infection were zero in 3 studies: Haiti, 2013 (95% CIs 0.0–1.4), Peru, 1994 (95% CIs 0.0–11.2), and Peru, 1991 (95% CIs 0.0–4.4); and 1.9% (95% CIs 0.1–10.57) in Senegal (2006). In 1969–1990, maternal mortality ranged from 0.0% (95% CIs 0.0–6.15) in Pakistan (1969) to 25.5% (95% CIs 18.8–34.0) in India (1969). Pooled maternal death rate for 1991–2013 was 0.2% (95% CIs 0.0–0.7) and for 1969–1990 5.0% (95% CIs 0.0–16.0), which is statistically different at p = 0.034. Studies in the 1991–2013 period showed no statistical heterogeneity according to *χ* ^*2*^ (p<0.05), and the Higgins *I* ^*2*^ for non-statistical heterogeneity was moderate at 59%; whereas for the 1969–1990 studies there was statistical heterogeneity (*χ* ^*2*^ (2df) = 26.9, p<0.001) and considerable non-statistical heterogeneity (Higgins *I* ^*2*^ = 93%). When compared to national estimates, the RR of maternal death from cholera was 4.5 in Senegal, however, not statistically significant (95% CIs 0.8–25.3, and p = 0.1305). For all four studies of 1991–2013, the RR is 0.79 (95% CIs 0.14–4.45) which is not significant (p = 0.8114).

## Discussion

From the four articles published during the 1991–2013 period, meta-analysis showed pooled estimates for fetal death of 7.9% (with no difference in risk of fetal demise by trimester), neonatal death of 0.8%, and maternal death of 0.2%. The pooled estimates from the 1959–1990 cohorts were 31.0% for fetal death, 6.4% for neonatal death, and 5.0% for maternal death. When compared to available recent national estimates for fetal, neonatal and maternal mortality, the two most recent studies from Senegal and Haiti suggest that there is an increased relative risk of fetal death of approximately 2 to 6 fold, but no increased relative risk of neonatal or maternal death.

Several issues should be considered when interpreting these risk estimates. Method of confirmation of pregnancy and estimation of gestational age were not mentioned in most studies, and when mentioned, it was through self-report. This could lead to measurement bias by underestimating the denominator (number of pregnant women), especially those in early first trimester, and result in inaccurate classification of pregnancies per trimester. Within the context of cholera outbreaks, it is assumed that all cases of diarrhea are caused by *V cholerae*. Except for the Pakistan cohort, which confirmed cholera cases by cultures, the other studies determined cholera mostly based on clinical signs and symptoms, which is an accepted WHO practice [9]. However, this can overestimate the number of pregnant women who actually had cholera and be source of measurement bias. Further, under-enumeration of deaths could occur. However, since these data emanate from health facilities, incorrect diagnosis of pregnancy and cholera, and under-enumeration of deaths are minimized, although the generalization of findings to all symptomatic pregnant women during a cholera outbreak who were not hospitalized cannot be done. Potential health facility-related biases include the admission of more severe cholera cases, the greater detection of cholera cases in pregnant women, the inclusion of cases living close the health facility, or the exclusion of women with cholera who died from cholera at home or under way to the health facility, or of women who could not go to the health facility and who did not receive appropriate treatment and for whom pregnancy outcomes could be more severe as a result. Results are based on a limited number of studies that differ in time, location, study methodology, and sample size. Except for the Haiti (n = 263) and India (n = 184) studies, the other studies had a smaller sample size (n = 33–84), which resulted in very small numerators of deaths and considerable variability in rates in some subgroups. Although there were insufficient studies to plot a frequency distribution to investigate possible publication bias, which may be due to non-publication of small studies with negative findings (e.g. studies with mortality rates that are similar to those observed in the population as a whole), it appears from Figs 2–4 that there are adequate numbers of studies with small rates, including a number of studies with zero rates.

Comparisons by period shows a significant higher absolute risk of fetal, neonatal and maternal death in 1969–1990 compared with 1991–2013, which is particularly marked for fetal death. This reduction in fetal, maternal and neonatal mortality may be explained by progress in cholera management over the past five decades. This includes not only improvement in standardized clinical protocols for cholera, but also in early warning and preparedness, continuous epidemiologic surveillance, good coordination among operational partners, community engagement, and measures to ensure sustainable safe water, sanitation and hygiene infrastructure, which can all contribute to the effective control of cholera outbreaks [8]. Except for the India study, all studies provide some level of information about their therapeutic approach to cholera (see Table 1): all administer more or less systematically different types and regimens of IV fluids and antibiotics, which is an accepted WHO practice [9]. Administration of oral rehydration solutions (ORS), a life-saving intervention from the 1960s, but only widely promoted by UNICEF and WHO in the late 1970s [21], was only mentioned in the articles from 1991 onward. ORS, along with other modern therapeutic and public health measures to control cholera outbreaks, could help explain the lower fetal, neonatal, and maternal mortality in more recent compared to earlier studies. The lower mortality in 1991–2013 compared to 1969–1990 cannot be explained by a difference in cholera biotypes as the outbreaks reported in all the reviewed articles were attributed to El Tor biotype.

Comparison between the third-trimester fetal death estimate from the Haiti cohort, which is the largest to date, and the national stillbirth estimates from 2010 strongly suggest that there was a higher risk of fetal death by almost six times in pregnant women affected by cholera (RR 5.7) compared to normative stillbirth data. Analysis of the Haiti cohort indicated that the strongest risk factors for fetal death were severe maternal dehydration and severe vomiting [6]. The 1991–2013 meta-analysed pooled rate for fetal death does not indicate a difference in risk of fetal death by trimester. This is surprising as in normal (non-cholera) circumstances, the risk of spontaneous abortion is known to be highest during the first trimester [22,23]. Such a higher risk would be expected to be compounded by the effects of cholera on pregnancy, but would not become apparent in the analysis if first-trimester pregnancies were under-enumerated.

The comparison of neonatal deaths in pregnant women with cholera from the Haiti and Senegal studies with their respective national NMR estimates matched by time period do not suggest a significant difference in risk of neonatal death. Although the individual national RRs for neonatal death are non-significant, the pooled RR of 0.28 for all four studies of 1991–2013 is significant at p = 0.0290 because of larger numbers. This suggests that neonatal mortality in pregnant women with cholera is lower than the national average. This may be because these women are under clinical care, including giving birth in hospitals, and thus this medical attention may confer an advantage for their children in the first 28 days. Long-term mortality and morbidity of cholera on children exposed to cholera during gestation was not well documented in the selected studies. Only the Pakistan cohort reported cases of seizures (n = 1) and severe spasticity (n = 2) among the 17 children who returned for follow-up within the first two years [16]. Longitudinal studies of newborns past the neonatal period are therefore needed to confirm these findings.

With regard to maternal death in pregnant women with cholera, the comparison of data from the Haiti and Senegal studies with their respective MMR estimates matched by time period do not suggest a significant difference in risk. There was no maternal death in the published Haiti cohort, while the overall case-fatality rate during the 2010 cholera outbreak in Haiti was 2.2% [10]. The meta-analysed pooled maternal death estimate of 0.2% for 1991–2013 remains within the margin of the international 1% cholera case-fatality rate benchmark.

Epidemic cholera is a disease of critical importance in many developing countries, as evidenced by the large outbreaks over the past decade [1]. The pooled meta-analysis of the most recent studies suggests that, during cholera outbreaks and using modern cholera management strategies, there is an absolute risk of fetal death of approximately 8% and a two- to six-fold increase in relative risk compared to national estimates. There is no indication of a significant increase in the relative risk of neonatal or maternal death compared to normative national data.

Despite the risk of adverse effects on mother, fetus, and neonate, there are currently no internationally-agreed guidelines and recommendations regarding the treatment of cholera in pregnancy. These meta-analysed estimates of cholera-affected pregnancy outcomes indicate the need to carry out further research on cholera and pregnancy, mobilize resources, and develop strategies to include pregnant women as an at-risk population into all policies, programmes and practices on cholera control.

## Supporting Information

S1 Checklist PRISMA 2009 checklist. (PDF)
